# 2239. Evaluating the Use of Cefuroxime for the Treatment of Outpatient Pyelonephritis at an Indian Health Service Hospital

**DOI:** 10.1093/ofid/ofac492.1857

**Published:** 2022-12-15

**Authors:** Vaishnavi Gopalakrishnan

**Affiliations:** Whiteriver Indian Hospital, Pinetop, Arizona

## Abstract

**Background:**

In 2015, a rural Indian Health Service Hospital found itself unable to use SMX/TMP due to high E. coli resistance rates. The stewardship team decided to include cefuroxime as a first line agent for outpatient pyelonephritis to have a fluoroquinolone sparing agent. Oral B-lactams are not typically recommended over SMX/TMP or fluoroquinolones for pyelonephritis, however there is sparse literature on the use of cefuroxime. This retrospective review aims to evaluate the efficacy of cefuroxime therapy for acute uncomplicated pyelonephritis over a five-year period.

**Methods:**

528 patients with a diagnosis of pyelonephritis from January 1st, 2015 to December 31st, 2019 were screened. Data collection included 30-day reinfection rates, emergence of ESBL isolates, duration of therapy, one time IV antibiotic administration, urine culture and susceptibility, increased risk (genitourinary abnormalities and diabetes), white blood cell count, and symptoms. Pediatric patients and patients requiring hospital admission were excluded.

**Results:**

264 patients were included and 188 patients received cefuroxime therapy. Baseline characteristics included 94.5% female patients, median age of 44 years, and average duration of therapy of 11.6 days. 95% of the isolates were E. coli. 31 patients had treatment failure defined as a return to the ED with 30 days for UTI. 25 of those patients received cefuroxime and of those, 20 had a one-time IV antibiotic dose. 10 patients with treatment failure had a history of ESBL after the encounter and of those 3 patients had cefuroxime therapy. 5 patients who had treatment failure with cefuroxime had diabetes and genitourinary abnormality.

**Figure:**
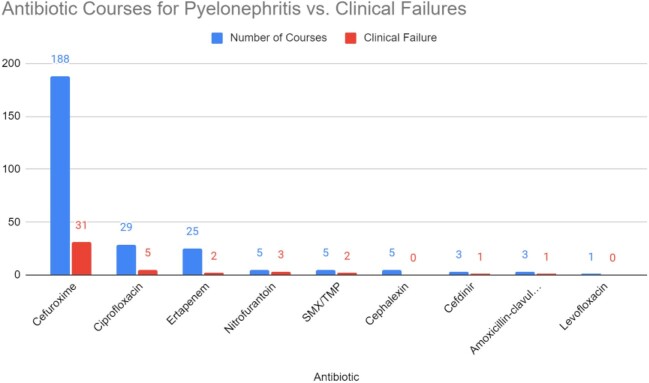

**Conclusion:**

This review suggests a lower rate of bacteriological cure of acute uncomplicated pyelonephritis with cefuroxime therapy with or without a one-time IV antibiotic dose with a cephalosporin or aminoglycoside.

**Disclosures:**

**All Authors**: No reported disclosures.

